# Initial impacts of the COVID-19 pandemic on Australian fisheries production, research organisations and assessment: shocks, responses and implications for decision support and resilience

**DOI:** 10.1007/s11160-023-09760-z

**Published:** 2023-03-02

**Authors:** Emily M. Ogier, David C. Smith, Sian Breen, Caleb Gardner, Daniel J. Gaughan, Harry K. Gorfine, Alistair J. Hobday, Natalie Moltschaniwskyj, Ryan Murphy, Thor Saunders, Mike Steer, James Woodhams

**Affiliations:** 1 Institute for Marine and Antarctic Studies, University of Tasmania, Hobart, Australia; 2 Centre for Marine Socioecology, Hobart, TAS Australia; 3 Commonwealth Scientific and Industrial Research Organisation, Hobart, TAS Australia; 4 Department of Agriculture and Fisheries, Brisbane, QLD Australia; 5 Western Australian Fisheries and Marine Research Laboratories, Department of Primary Industries and Regional Development, Government of Western Australia, North Beach, WA Australia; 6 Fisheries Management and Science Branch, Victorian Fisheries Authority, Queenscliff, VIC Australia; 7 Department of Primary Industries, Sydney, NSW Australia; 8 Australian Fisheries Management Authority, Canberra, ACT Australia; 9 Department of Industry, Tourism and Trade, Fisheries Division, Darwin, NT Australia; 10 Aquatic and Livestock Sciences Division, South Australian Research and Development Institute, Adelaide, SA Australia; 11 Australian Bureau of Agricultural and Resource Economics and Sciences, Canberra, ACT Australia

**Keywords:** COVID-19 pandemic, Systemic shock, Disruption, Fisheries production, Fisheries monitoring and assessment

## Abstract

Australia’s fisheries have experience in responding individually to specific shocks to stock levels (for example, marine heatwaves, floods) and markets (for example, global financial crisis, food safety access barriers). The COVID-19 pandemic was, however, novel in triggering a series of systemic shocks and disruptions to the activities and operating conditions for all Australia’s commercial fisheries sectors including those of the research agencies that provide the information needed for their sustainable management. While these disruptions have a single root cause—the public health impacts and containment responses to the COVID-19 pandemic—their transmission and effects have been varied. We examine both the impacts on Australian fisheries triggered by measures introduced by governments both internationally and domestically in response to the COVID-19 pandemic outbreak, and the countermeasures introduced to support continuity in fisheries and aquaculture production and supply chains. Impacts on fisheries production are identified by comparing annual and monthly catch data for Australia’s commercial fisheries in 2020 with averages for the last 4–5 years. We combine this with a survey of the short-term disruption to and impacts on research organisations engaged in fisheries monitoring and assessment and the adaptive measures they deployed. The dominant impact identified was triggered by containment measures both within Australia and in export receiving countries which led to loss of export markets and domestic dine-in markets for live or fresh seafood. The most heavily impact fisheries included lobster and abalone (exported live) and specific finfishes (exported fresh or sold live domestically), which experienced short-term reductions in both production and price. At the same time, improved prices and demand for seafood sold into domestic retail channels were observed. The impacts observed were both a function of the disruptions due to the COVID-19 pandemic and the countermeasures and support programs introduced by various national and state-level governments across Australia to at least partly mitigate negative impacts on harvesting activities and supply chains. These included protecting fisheries activities from specific restrictive COVID-19 containment measures, pro-actively re-establishing freight links, supporting quota roll-overs, and introducing wage and businesses support packages. Fisheries research organisations were impacted to various degrees, largely determined by the extent to which their field monitoring activities were protected from specific restrictive COVID-19 containment measures by their state-level governments. Responses of these organisations included reducing fisheries dependent and independent data collection as required while developing strategies to continue to provide assessment services, including opportunistic innovations to harvest data from new data sources. Observed short run impacts of the COVID-19 pandemic outbreak has emphasised both the vulnerability of fisheries dependent on export markets, live or fresh markets, and long supply chains and the resilience of fisheries research programs. We suggest that further and more comprehensive analysis over a longer time period of the long-run impacts of subsequent waves of variants, extended pandemic containment measures, autonomous and planned adaptive responses would be beneficial for the development of more effective counter measures for when the next major external shock affects Australian fisheries.

## Introduction

Commercial fisheries producers and fisheries research organisations are used to responding to variability in fish stocks and availability (Badjeck et al. 2010; Barbeaux et al. 2020; Fisher et al. 2021; Shelton et al. 2011), as well as additional pressures introduced through changes to management settings or short-term single events, such as marine heatwaves (Cheung et al. 2020; Smith et al. 2021) and temporary loss of markets (for example, the SARS outbreak in 2002–2004—see Keogh-Brown et al. 2010). The outbreak of severe acute respiratory syndrome coronavirus 2 (SARSCoV-2), causing coronavirus disease 2019 (hereafter COVID-19), triggered a pandemic in early 2020 and exposed fisheries globally to a crisis event which has triggered a series of cascading social and economic shocks, causing a range of short and longer-run disruptions that were unprecedented in terms of severity, endurance and their systemic nature (FAO 2020; OECD 2020).

Commercial fishing harvest sectors and fisheries research organisations have been exposed to both direct disease impacts, as well as to subsequent economic and social shocks arising from government-led pandemic response necessary to protect public health (Vecchio et al. 2022). These experiences have been examined in a range of case studies of commercial fisheries production (Asante et al. 2021; Coll et al. 2021; Smith et al. 2020) and fisheries research organisations and programs (Huveneers et al. 2021; Link et al. 2021) in developed economies. Notably, geopolitical and regional factors have had a large influence on the transmission of and response to these COVID-19 induced shocks. This is illustrated by the disruptions to labour supply for seafood producers due to COVID-19 illness reported globally (Sorensen et al. 2020), compared with the relatively limited, short-term exposure of Australia’s fisheries to this type of shock due to the different extent of disease prevalence across populations (Ogier et al. 2021).

The most notable disruptions to fisheries sectors in developed economies in the initial period of pandemic outbreak have been those primarily triggered by necessary government-led pandemic containment measures (e.g., physical distancing, movement restrictions, border closures). These measures disrupted fisheries production and scientific activity by restricting operational activities and movement of product and people (Asante et al. 2021; Sorensen et al. 2020), limiting supply of inputs to both fish harvesting and research activities (Chang et al. 2022), de-coupling freight links to markets (Carlson, et al. 2021), and dampening seafood demand (Fernandez-Gonzalez et al. 2021; Giannakis et al. 2020). Further knock-on effects identified include negative feedbacks on fisheries assessment and harvest settings arising from limited supply of fishery dependent data due to reduced fishing effort (Haas et al. 2021; Link et al. 2021; Plagányi et al. 2021).

Mitigating factors have included a range of short-term countermeasures introduced rapidly by governments during the initial pandemic outbreak period, including those to provide cash-flows to ensure business continuity and stimulus to maintain consumer demand, through to those to subsidise freight services to re-establish disrupted, largely export supply chains, and to incentivise investment in less vulnerable supply chains. Responses of seafood industries have included developing or improving less vulnerable domestic supply chains and markets given the downward effects on seafood trade due to international border restrictions (Stoll et al. 2021; White et al. 2021).

Short-term adaptive responses by research organisations reveal the extent of organisational capacity for not only adaptive risk management (Santora et al. 2021) but also for innovation (Link et al. 2021). The COVID-19 shocks observed presented novel challenges which—in turn—led to conditions of exceptionalism and an enabling environment for novel and experimental strategies to be trialled (Huveneers et al. 2021; Kemp et al. 2020). Historically, drivers of innovation in fisheries science programs have included the need to account for long-term trends (for example, climate-driven changes) or expanded scope of assessment in response to social change and have tended towards increasing complexity and lags in implementation (Bradley et al. 2019; Gorospe et al. 2016). Globally and more generally, rapid adaptive and innovative responses to the COVID-19 crisis by fisheries research organisations and management agencies—including alternative methods of monitoring and assessment—are becoming institutionalised as pay-offs are recognised (Kemp et al. 2020; Santora et al. 2021).

Our study aimed to describe the types and extent of shocks and disruptions stemming from pandemic containment measures for the period of January 2020–June 2020, and the subsequent impacts to Australia’s commercial fisheries production and fisheries research organisations’ monitoring and assessment activity. Our analysis also aimed to take account of the short-term countermeasures and adaptive responses implemented rapidly during this period and the extent to which they were observed to mitigate disruptions. We do this through analysis of the introduction of pandemic containment measures implemented by Australia’s federal, state and territory jurisdictions in major export market countries and in Australia. We analyse levels of fisheries production using annual production data and monthly catch data provided for fisheries and species. We describe immediate adaptive responses of fisheries research organisations identified through survey methods and drawn from public records. Using Australia as a single country case, we are nonetheless able to compare between Australia’s jurisdictions because of differences in disease exposure and containment measures. In addition, the absence of some other potentially confounding factors (e.g., socioeconomic disparity) was limited compared to many other countries, strengthening the ability to compare the role of jurisdictional government measures as a factor influencing impacts arising from COVID-19 induced shocks.

Furthermore, examination of the types of disruptions and the responses by Australian fisheries producers and science organisations presents the opportunity to identify features of fisheries science activity and organisations that enable resilient science provision under crisis conditions to support fisheries management. The questions we address in this paper are as follows:

- What types of disruptions have been transmitted to commercial fisheries production and fisheries research organisations by government-led pandemic containment measures, and what were the short-term impacts?
- What adaptive measures and risk management strategies which support the continuity of fisheries monitoring and assessment have fisheries research organisations adopted, and with what implications for innovation and future resilience to systemic shocks?

## Background

Total annual production for Australian commercial fisheries was approximately 180,000t during 2019/20, and had a value of AU$1.55 billion (Steven et al. 2021). Australia’s fisheries are extremely diverse, operating in tropical to sub-Antarctic ecosystems. They are managed on a stock basis by the Commonwealth (Federal), State and the Northern Territory governments, although there are inter-jurisdictional arrangements in place for stocks which straddle waters managed by multiple states and/or the Commonwealth (Vince et al. 2015). Both imports and exports play a major role in Australia’s seafood markets. In 2019/20 approximately 62% of the volume of seafood consumed in Australia was imported, while the value of the Australian-produced seafood which was exported was approximately 50% of the total value of Australia’s seafood (Steven et al. 2021).

While the focus of this paper is on the effects of the initial COVID-19 pandemic outbreak on commercial fisheries, Australia has a large and increasing aquaculture sector valued at over AU$1.6 billion in 2019/20 (Steven et al. 2021). There are also substantial recreational fisheries (Henry and Lyle 2003) and culturally-significant Indigenous fisheries (AIATSIS 2022; Saunders et al. 2016; Schnierer et al. 2016).

Undertaking an analysis of COVID-19-induced impacts on commercial fisheries and associated monitoring, research and assessment required agreement and collaboration across Australia’s fisheries jurisdictions and agencies. The National Research Providers Network for Fisheries and Aquaculture (RPN), established in 2010 (FRDC 2010), provided this mechanism. The RPN is a cross-agency government level committee that develops coordinated responses to fisheries and aquaculture research priorities. Membership also includes the Fisheries Research and Development Corporation, the Integrated Marine Observing System and Oceanwatch and the Australian Society for Fish Biology.

## Methods

### Timeline of government-led public health protection measures, disruptions and countermeasures

Government-led public health measures introduced in Australia and in major export-destination countries, such as China, were identified using two chronologies of COVID-19 related public announcements by State, Territory and Federal Governments for the period 01 January to 30 June 2020 collated by the Parliament of Australia (Storen et al. 2020; Campbell et al. 2021). These datasets included announcements of domestic pandemic response measures by these governments, as well as countermeasures. Information on containment measures implemented in China was drawn from a database of epidemic trends and control measures during the first wave of COVID-19 in mainland China (Fu et al. 2021). For the purposes of comparative analysis, the public health measures announced were categorised by: type of measure; severity of the measure; and, month(s) of implementation and duration. Types of measures included interstate and international border restrictions, physical distancing, and intra-state movement restrictions. Severity of measure was categorised as either ‘High’ or ‘Low’ using the tiered systems of measures deployed by the various governments to distinguish between levels of measures implemented (1) during periods of growth in daily cases of infection (i.e. high level of severity) and (2) during the easing of restrictions as outbreaks were contained (i.e. low level of severity). Measures implemented before the 15th day of a given month were classed as present for the full month, while measures implemented after the 15th day were classed as not present. The exception to this rule was where a ‘High’ severity measure was introduced after the 15th day of a given month, in which case the whole month was categorised as ‘Low’ severity to reflect the assumption that some level of impact would have been experienced due to the introduction of the health measure.

### Rapid assessment of impacts on fisheries production

National annual commercial fisheries statistics (Steven et al. 2021) were examined for the period 2010/11 to 2019/20 to see if there was a detectable variation in quantity and/or value in 2019/20. Annual statistics for each jurisdiction were examined to ascertain if different responses were evident. Given that annual landings might disguise effects on individual fisheries, monthly catch data were also examined for the major fisheries and species for each jurisdiction for the first six months of 2020. Monthly catches are often quite variable, so monthly catches in 2020 were compared to monthly catches in the previous 4–5 years.

Fisheries or species for which monthly catches for January 2020–June 2020 were below the range of the previous five years were identified. Discussions were had with RPN representatives of each jurisdiction to ascertain reasons and insights as to the factors contributing to lower catches and prices in these cases. Factors identified included disruptions to export and domestic markets and associated supply chains triggered by government measures to contain the COVID-19 pandemic in both export market countries and within Australia, as well as other impacts such as labour shortages arising from domestic COVID-19 public health protection measures (i.e. physical distancing, movement restrictions). While these COVID-19 linked factors were a major cause in many fisheries, other factors were also evident, such as environmental conditions and changed management measures. Data custodians from each fisheries research organisation were asked to sign-off on the results, interpretation and level of reporting that was possible, given the confidentiality provisions regarding reporting of some species and fisheries. In some cases, it was not possible to report on individual species or fisheries, and in other cases fisheries or species are combined to allow reporting (for example, abalone species).

### Inventory of impacts on and responses of fisheries research organisations

The RPN met three times between March and June 2020 and again in September 2020. The primary aim of these meetings was for research organisations and management agencies to report and provide updates on changes to activities due to government directions in response to the COVID-19 pandemic and discuss constraints and the potential for shared services if these were required. Constraints and implications covering two main areas were tabulated for each agency:

- Current arrangements, procedures, and guidelines – capturing specific impacts of jurisdictional public health measures such as lockdowns, working from home, field work and travel, and project delivery.
- Implications for fisheries monitoring, assessment and provision of management advice – this covered commercial fisheries, recreational fisheries, Indigenous fisheries and aquaculture. Topics included data collection, assessment and provision of management advice including harvest strategies.

Organisational responses to these constraints and implications were summarised across jurisdictions.

## Results and discussion

### Government-led pandemic responses affecting Australian fisheries

Markets for Australian seafood were impacted, severely in some cases, by government-led pandemic containment measures in Australia and in export receiving countries. China’s government was the first to introduce physical distancing and movement restrictions to contain the outbreak of the COVID-19 epidemic in late January 2020 (Table 1). These measures had a significant dampening effect on demand for imported Australian live seafood as it triggered the cancellation of Chinese Lunar New Year celebrations in many cities and provinces. The Australian Government’s subsequent closures of its international borders (Table 1) triggered further disruption due to the loss of seafood demand from inbound tourism markets, and the loss of outbound air freight capacity to export markets.

**Table 1:**
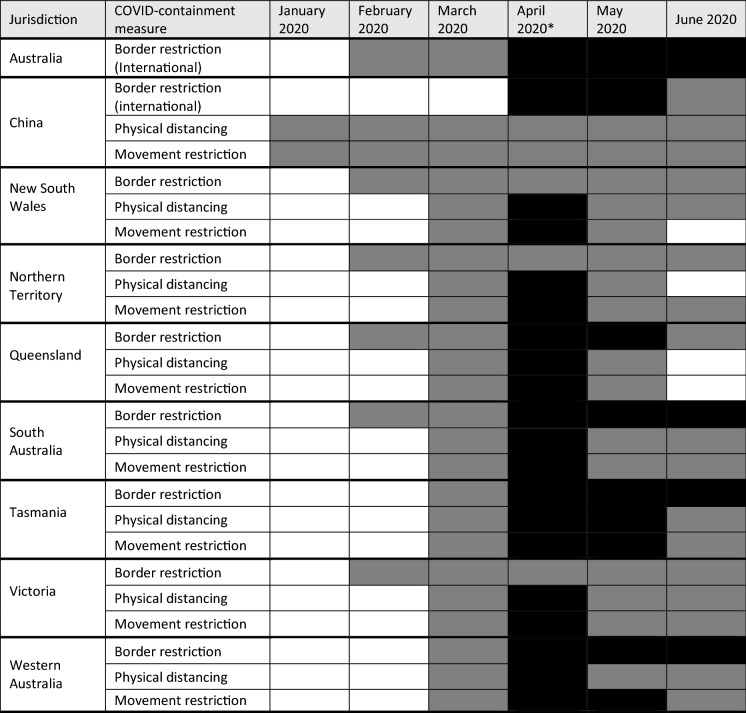
Presence and degree of government-led COVID-19 containment measures implemented by China and Australia’s national governments, and by Australian state and territory governments, January 2020—June 2021, in response to the COVID-19 pandemic outbreak. White shade indicates no measure present for 50% or more of the month; Grey shade indicates presence of Low-level measures for > 50% of month OR High-level for < 50% of month; Black shade indicates presence of High-level measures for 50% or more of the month. *Source*: Fu et al. 2021; Storen et al. 2020; Campbell et al. 2021

*Introduction of Australian Government countermeasures to allow workers in fisheries production and processing to be excepted from physical distancing requirements; and to support continuity of international supply chain

Similar types of pandemic containment measures were implemented in all Australian jurisdictions across the periods February–June 2020 by the Commonwealth (Federal, State and Territory governments (Table 1), including State border closures. These measures triggered disruptions to domestic fisheries production through labour shortages and restrictions on seafood processing activity initially, and to domestic seafood demand through, loss of long-haul freight services initially, restrictions on food service (in particular, dine-in), and heightened demand through domestic retail markets. For the initial February-April period, the timing and severity of these measures and disruptions was fairly uniform. This is attributable to the formation of the National Cabinet in mid-March, which resulted in a centrally-coordinated and more consistent set of pandemic containment responses. In contrast, in May and June there was a greater degree of variation observed in the severity and longevity of containment measures. This had flow on impacts on how individual research agencies were disrupted and their capacity to respond, which are outlined below.

Two countermeasures introduced by the Federal Government in 27 March and 01 April, respectively, were designed to partly mitigate the disruptions of these pandemic containment measures to domestic food production sectors. Agricultural workers and businesses (including fisheries and aquaculture) were re-classified by the National Cabinet—an inter-jurisdictional decision-making body formed in response to the COVID-19 pandemic outbreak—as ‘essential’, and therefore exempt from physical distancing, domestic border and movement restrictions when carrying out this work. On-board fisheries observers and fisheries research organisation staff were not included in this countermeasure. The International Freight Assistance Mechanism (IFAM) was introduced as a temporary, targeted, emergency support measure by the Australian Government to keep global air links open for export sectors in response to the ongoing effects of the COVID-19 pandemic. Australian seafood export-facing sectors were eligible for this mechanism.

During the first part of 2020 Australia’s fisheries management agencies implemented several additional countermeasures in response to the impacts of COVID-19 on the Australian fishing industry. These included deferring, or waiving fishing licence fees and levies, and rolling over uncaught quota into the following year for specific fisheries, all of which required management flexibility to be exercised. The Australian Fisheries Management Forum (Heads of Agencies) also met to discuss and compare the extent of disruption and available management agency and national government responses.

### Impacts on total commercial fisheries production and value

At the national level, total annual fisheries production was not impacted by the COVID-19 pandemic however impact on total annual fisheries value is evident, as is impact on production and volume for specific species groups (Table 2). The total quantity of crustaceans in 2019/20 was the lowest reported during this period and the total value was the lowest since 2012/13. The quantity of molluscs reported in 2019/20 was the lowest since 2015/16, and 2014/15 by value. This in part reflected COVID-19 disruptions to supply chains and demand for crustacea (rock lobster) and mollusc (abalone) fisheries that were predominantly export-market oriented. For example, the value of total crustacea and mollusc exports in 2019/20 was the lowest in seven years (Steven et al 2021). For rock lobsters and abalone, the quantity exported was considerably lower than the previous 10 years. Conversely, production of finfish in 2019/20 was the highest reported during this period in both quantity and value, which is likely to partly reflect the domestic retail market orientation of major finfish fisheries.

**Table 2:** Total fisheries production (quantity and value) for the period 2010/11 to 2019/20 for species groups. ^p^ refers to the provisional status of this data. Source: Steven et al. 2021

	2010–11	2011–12	2012–13	2013–14	2014–15	2015–16	2016–17	2017–18	2018–19	2019-20^p^
*Quantity (tonnes)*										
Finfish	110,504	113,803	108,700	105,083	104,666	126,494	115,485	120,405	117,375	125,769
Crustaceans	39,288	33,014	32,996	37,114	35,979	35,114	36,627	35,182	34,949	30,518
Molluscs	15,104	12,248	15,410	11,020	11,556	12,394	13,598	15,772	13,335	12,478
Total	**165,163**	**159,294**	**157,283**	**153,504**	**152,432**	**174,246**	**166,016**	**173,434**	**178,723**	**179,261**
*Value (AU$’000)*										
Finfish	410,871	452,304	449,524	414,951	431,024	516,271	489,107	500,130	510,998	560,225
Crustaceans	705,245	664,510	718,619	924,222	1,007,442	1,055,947	1,049,388	1,075,764	1,071,369	826,628
Molluscs	205,409	181,334	198,358	173,414	176,022	176,315	202,321	214,339	206,047	175,622
**Total**	1,322,783	1,305,490	1,367,401	1,513,742	1,615,670	1,749,454	1,742,087	1,793,211	1,794,992	1,581,141

At the state and territory level, impacts on fisheries production and value varied across jurisdictions with annual data in 2019/20 signalling effects of disruptions induced by the COVID-19 pandemic for some, but for others the impacts and attribution were less clear (Table 3). In Western Australia, 2019/20 was the lowest volume of production reported since 2014/15 and the lowest value since 2012/13. South Australia’s production in 2019/20 was the lowest since 2014/15 by quantity and the lowest since 2013/14 by value. The reported Victorian catch in 2019/20 was the lowest in the time series. A combination of commercial netting removals from bays and estuaries, and variable catch patterns among line and offshore seining due to low profitability and sporadic abundance contributed to this, but there was no clear trend or signal by value. For 2019/20, Queensland’s production was reported as the lowest volume and value in the time series. However, a number of significant fisheries reforms were well underway in early 2020, potentially confounding any production or value related impacts from COVID-19 on Queensland’s commercial fishing sector. The Tasmanian landings in 2019/20 were slightly lower than 2018/19 but still higher than the previous years, however the value was the lowest since 2012/13. The production volume reported for 2019/20 for the Northern Territory was the lowest since 2014/15 but the value was in the range of previous years. There was not an apparent impact of COVID-19 based on reported NSW catch and value in 2019/20. The Commonwealth quantity reported for 2019/20 was the highest in the time series and the value highest since 2015/16.

**Table 3:** Total fisheries production (quantity and value) for the period 2010/11 to 2019/20 for each Australian jurisdiction. Source: Steven et al. 2021

Jurisdiction	2010–11	2011–12	2012–13	2013–14	2014–15	2015–16	2016–17	2017–18	2018–19	2019-20^a^
*Quantity (tonnes)*										
New South Wales	14,201	13,151	12,390	13,614	12,024	11,742	10,574	11,312	12,451	11,642
Victoria	5549	5401	4504	4356	3802	4476	4845	3961	4222	2928
Queensland	22,704	21,840	25,025	20,945	20,301	19,269	19,855	19,853	17,425	16,927
South Australia	43,132	46,561	44,215	41,867	45,155	50,683	49,487	52,833	49,145	48,422
Western Australia	22,764	18,348	18,889	19,007	19,804	20,513	22,321	22,846	20,421	20,031
Tasmania	4,662	4,732	7,338	5,476	4,139	4,680	3,620	5,314	15,733	12,803
Northern Territory	5315	6087	5805	5331	5340	6110	6722	6224	5926	5857
Commonwealth	46,836	43,174	39,118	42,907	41,868	56,773	48,592	51,090	53,400	60,652
*Value (AU$'000)*										
Commonwealth	320,811	308,244	317,814	340,453	350,276	438,829	403,350	390,078	436,971	438,379
New South Wales	79,149	77,265	80,694	92,479	89,484	91,082	89,305	99,501	106,234	104,508
Northern Territory	32,442	34,104	34,090	30,359	31,071	34,894	43,860	47,825	38,538	40,534
Queensland	194,739	185,616	196,213	191,334	182,209	175,897	192,832	180,199	158,942	155,408
South Australia	195,440	208,928	198,105	210,410	240,204	264,653	253,107	264,049	276,686	231,829
Tasmania	163,053	155,982	153,869	176,947	175,265	182,349	175,935	194,317	187,411	160,350
Victoria	51,258	55,474	55,745	54,840	58,742	57,810	54,362	62,770	71,596	62,321
Western Australia	285,890	279,877	330,872	416,919	488,420	503,939	529,336	554,472	518,613	387,811

^a^Refers to the provisional status of this data

The negative impact on production volume and value is likely to reflect the different contributions of crustaceans and molluscan fisheries to total production volume and value in each jurisdiction, as these species groups were highly exposed to export market shocks. In contrast, the increased catch in Commonwealth fisheries may reflect the increased landings of blue grenadier and orange roughy in June, as well as positive domestic market conditions because of COVID-19 induced disruptions to imported finfish.

### Impacts on monthly commercial fisheries production for selected species and fisheries

Catch volumes of a number of Australian fisheries were impacted in the short-term by the disruptions to markets and supply chains triggered by the outbreak of the COVID-19 pandemic. Figures presented below show monthly catch volumes for 2020 compared to the range of monthly catches for the period 2015–2019 or 2015/16–2018/19 for fisheries and species in each jurisdiction where monthly catches were outside the range (i.e., lower than) of previous years.

#### Commonwealth (Federal)

**Fig. 1:**
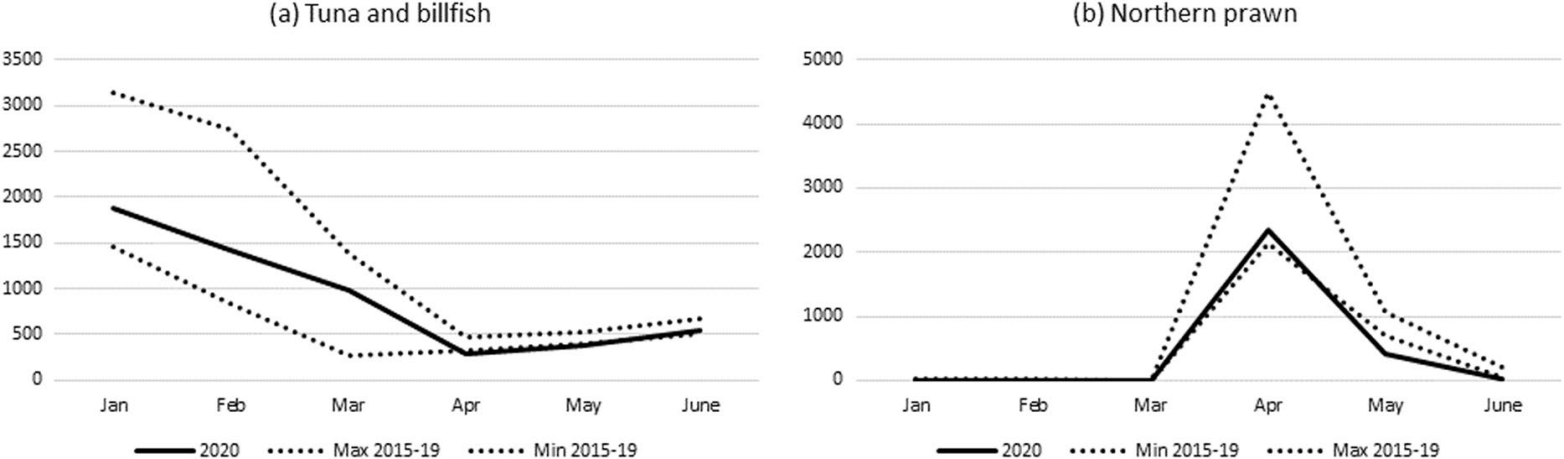
Monthly catch (tonnes) of (**a**) tunas and billfishes and in (**b**) Northern Prawn Fishery in 2020, compared with the range of monthly catches for 2015–2019. *Source*: Australian Fisheries Management Authority

The tuna and billfish fisheries have a high international exposure as much of their product is exported. The fisheries in aggregate are shown below (Fig. 1a). Overall, catches were slightly below the range of the previous five years during April and May although several factors were involved. The Southern Bluefin Tuna Fishery had slightly lower catches during February and made up some in March. This appears to reflect environmental factors influencing the timing of fish capture rather than COVID-19 induced disruptions. Western Billfish and Tuna Fishery catches were much lower during 2020, driven by a lack of international air freight availability to distribute fish (see Table 1) so the focus was on the local market. However, catches are small and did not contribute substantively to the total.

Monthly catches in 2020 for the Eastern Billfish and Tuna Fishery were within the range of the previous five years. These aggregate figures, however, mask differences at the species level. Catches were lower for bigeye tuna and particularly for broadbill swordfish—for the latter, reflecting a disruption of access to markets on the east coast of the US. This was offset by increased catches of albacore, in part reflecting increased freezing capacity with funding assistance through a Federal Government grant as part of a COVID-19 economic support package.

In the Northern Prawn Fishery banana prawn catches were lower during May and June but again environmental factors, such as low rainfall, are thought to be the main reason (Fig. 1b). Total Southern and Eastern Scalefish and Shark Fishery catches were lower for some species and a slightly lower total monthly catch in February and May. The availability of crew was reported as an issue during some of 2020, due to movement restrictions (see Table 1). However, overall, the total catch was higher due to increased catches of blue grenadier and orange roughy in June.

#### New South Wales

**Fig. 2:**
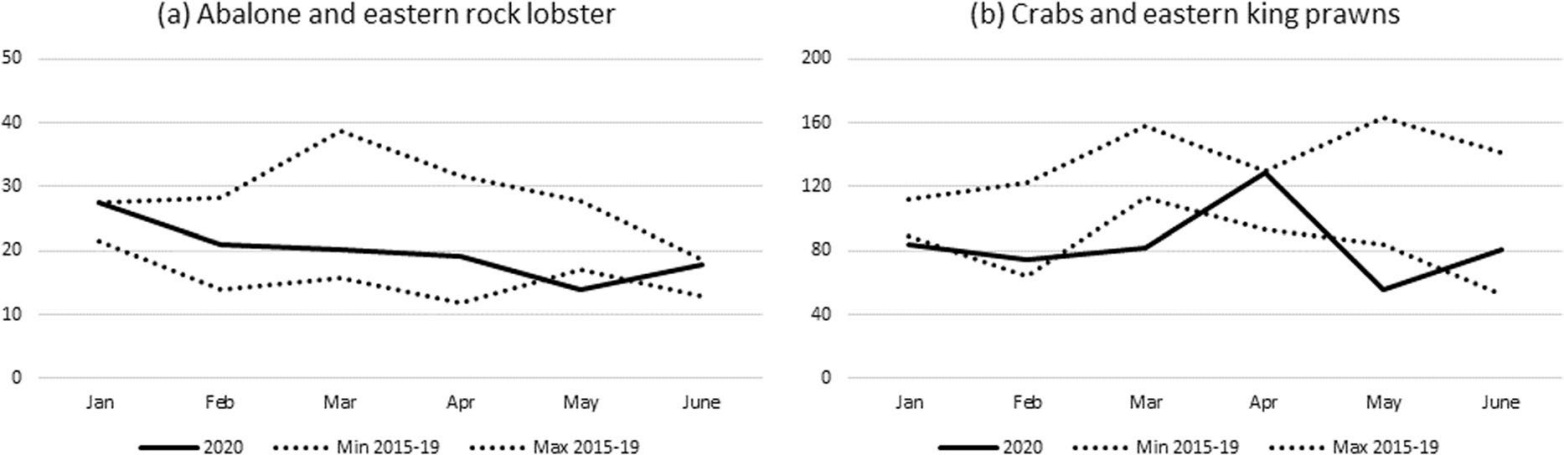
Monthly catch (tonnes) for (**a**) abalone and eastern rock lobster and (**b**) crabs and eastern king prawn in 2020, compared with the range of monthly catches for 2015–2019. *Source*: NSW Department of Primary Industries

The impacts of COVID-19 on wild fisheries production were relatively modest. As with other jurisdictions abalone catches were impacted during January to May. However, unlike with southern rock lobster, monthly catches of eastern rock lobster were all within the range of catches during the previous five years (Fig. 2a, combined fisheries).

Eastern king prawn catches were lower than the previous five-year range in March and May but higher in April. This reflects a COVID-19 impact but also an industry response through identifying new markets (Fig. 2b). The impact of COVID-19 on crab species was variable. There was a direct effect on blue swimmer (January to June) and mud crabs (March to June) due to loss of demand for live product from dine-in food service markets, due to physical distancing measures (see Table 1). For the latter there were also COVID-19 impacts on supply chains through disruptions to freight service availability. However, while catches of spanner crabs were lower in 2020, this was primarily due falling catch rates and higher costs of capture, with some additional impacts associated with flood events in early 2020, and loss of international markets due to COVID-19.

There were no major impacts on other species. For all other species combined, catches were lower than the five-year range in several months during 2019/20 but this is attributed to management intervention rather than COVID-19-induced impacts alone.

#### Northern Territory

**Fig. 3:**
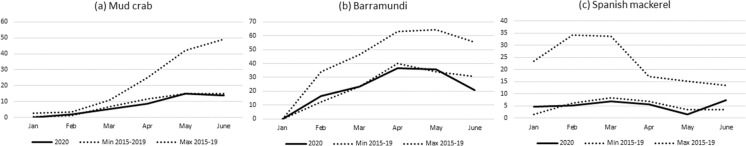
Monthly catch (tonnes) for (**a**) mud crab, (**b**) barramundi and (**c**) Spanish mackerel in 2020, compared with the range of monthly catches for 2015–2019. *Source*: NT Department of Industry, Tourism and Trade

Monthly mud crab catches were lower than the range of the previous five years from March onwards (Fig. 3a). The price dropped substantially due to loss of domestic markets (e.g. loss of demand for live product from dine-in food service markets, and competition from other products such as rock lobster that lost their international markets, arising from physical distancing measures and international border closures, see Table 1). Other impacts included workforce shortages due to border restrictions (see Table 1), restrictions on access to Aboriginal land, and reduced air freight service limiting live export.

The lower catches in the Barramundi Fishery were primarily due to successive poor wet seasons resulting in a lower stock biomass (Fig. 3b), although the commercial sector would have been slightly impacted by transactional costs arising due to COVID-19 health measures, as would all fisheries initially. Similarly, Spanish mackerel catches (Fig. 3c) were lower for reasons other than COVID-19 induced impacts and was probably associated with a reduction in stock size probably due to lower recruitment in 2016, the same year a marine heatwave occurred across northern Australia (Benthuysen et al. 2018).

For all the other fisheries combined, catches were lower during April. In the Demersal Fishery, catches were down in April due to short-term COVID-19 related shortages in crew, maintenance services and supplies because of border restrictions.

#### Queensland

With the release of the *Sustainable Fisheries Strategy 2017–2027*, its implementation and discussion of significant fisheries structural reforms underway in early 2020, it is likely these confounded any production or value related impacts from COVID-19 on Queensland’s commercial fishing sector. Reforms being considered included introduction of individual transferable quota and spatial management and mandatory vessel tracking implemented on all commercial vessels. However, the impact of COVID-19 disruptions through international border closures (see Table 1) was evident in those fisheries which relied on high value, live export markets.

**Fig. 4:**
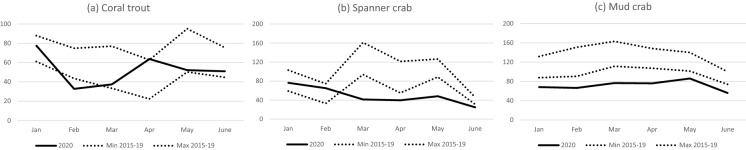
Monthly catch for (**a**) coral trout, (**b**) spanner crab and (**c**) mud crab in 2020, compared with the range of monthly catches for 2015–2019. *Source*: QLD Department of Agriculture and Fisheries

The coral trout catch was low in February (Fig. 4a) and the spanner crab catch was low from March to June (Fig. 4b). Both fisheries lost export markets, resulting in a reliance on the competitive domestic market during a time of extended lockdowns in the capital cities. Mud crab catches were down from January to June (Fig. 4c), primarily due to reduced domestic markets similar to that reported for the Northern Territory.

Barramundi catches were slightly lower from April to June, but this was primarily management related. The catch of the East Coast Prawn Fishery was slightly lower in March 2020, but this is not considered a result of COVID-19-induced impacts. There were limited impacts on both catches and prices in other fisheries including the Tropical Rock Lobster Fishery.

#### South Australia

**Fig. 5:**
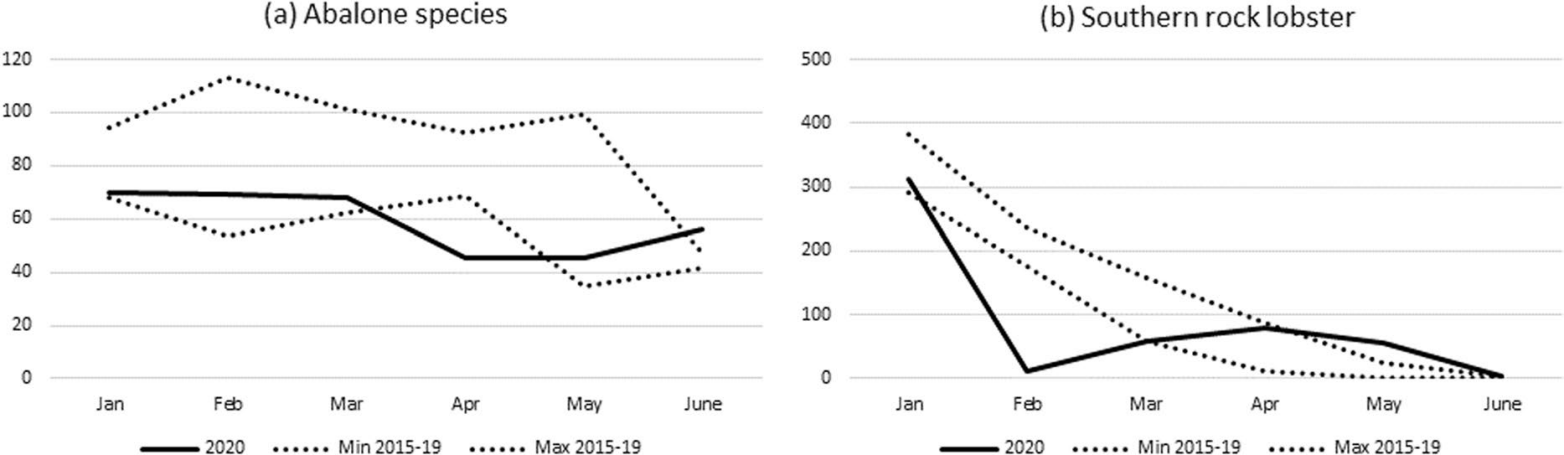
Monthly catch (tonnes) for (**a**) abalone species and (**b**) southern rock lobster in 2020, compared with the range of monthly catches for 2015–2019. *Source*: South Australian Research and Development Institute

The blacklip abalone catch was very low during April (Fig. 5a) reflecting limited international markets due to COVID-19 induced international border closures and low freight capacity (see Table 1). In addition, the timing of the catch also had an influence as the western zone fishery caught > 50% of the 2020 calendar year quota in January and February. Greenlip abalone catches were slightly lower compared to previous years in March and April (also January). New seasonal closures from January to March may have contributed to this in some areas. It was also reported that there was under catching quota in one zone. Consequently, the COVID-19 induced impacts were not as high as for blacklip abalone. The southern rock lobster catch was very low during February 2020 due to COVID-19 induced impacts (Fig. 5b) but there appeared to be some recovery in April and May as international markets reopened and access was increased. A similar pattern of low catches in February was also seen in Victoria and Tasmania.

The blue swimmer crab catch was low in April. This was reported as being due to a combination of limited dine-in food service markets and a consequence of a high percentage of the quota having already been caught. The aggregated catch of “other crustaceans” (including blue swimmer crabs, prawn species combined, and giant crab) was also very low during June. However, it is unclear wherever this is COVID-19 related or due to not all the data being available due to confidentiality issues (or a combination of both). Monthly catches for all other coastal and marine species combined were slightly lower during March to April but much of this was probably due to the closure of most snapper fisheries.

#### Tasmania

**Fig. 6:**
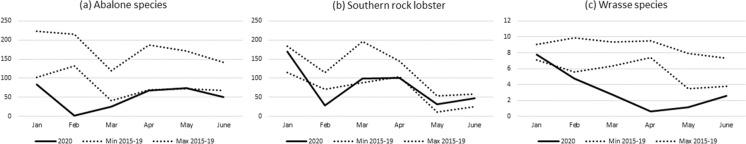
Monthly catch (tonnes) for (**a**) abalone species, (**b**) southern rock lobster and (**c**) wrasse species in 2020, compared with the range of monthly catches for 2015–2019. *Source*: Institute for Marine and Antarctic Studies, University of Tasmania

Monthly catches for abalone (species combined) in 2020 were lower than the range of the previous five years for the first four months of the year, particularly February, reflecting COVID-19 induced international border closures and impacts on exports markets (Table 1, Fig. 6a). However, there was also a cut to the TAC which also contributed to lower catches during 2020. Similar to South Australia and Victoria, southern rock lobster monthly catches were very low in February, and also March and April, as international markets were disrupted by COVID-19 induced impacts but there appeared to be some recovery in later months (Fig. 6b).

Wrasse catches were lower during February to June (Fig. 6c). This was due to physical distancing measures and border restrictions (see Table 1) severely restricting the domestic dine in food service sector, impacting the live fish market (banded morwong catches were also lower). Similar patterns were reported for Victoria’s Wrasse Fishery. Monthly catches of giant crab were lower during April to June, however catches can be quite variable. Giant crabs are mainly sold live into Asian restaurants in Australia. Reduced export demand for southern rock lobsters led to more lobsters being supplied at a lower, more competitive price into Australian markets. These species tend to be close substitutes so demand for giant crab was reduced.

Monthly catches of other species were generally lower than the range of the previous five years from January to June. There are clearly some COVID-19 induced impacts through loss of markets and dampening of demand. However, there are a range of other factors affecting production. For example, several species have a downward catch trajectory spanning > 10 years, mainly driven by competition from aquaculture or imported substitutes.

#### Victoria

**Fig. 7:**
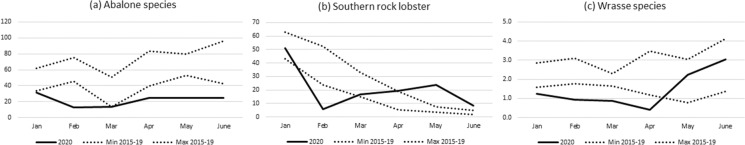
Monthly catch (tonnes) for (**a**) abalone species, (**b**) southern rock lobster and (**c**) wrasse species in 2020, compared with the range of monthly catches for 2015–2019. *Source*: Victorian Fisheries Authority

Monthly abalone (species combined) catches were generally lower throughout 2020 but particularly (in relative terms) during February and from May to June (Fig. 7a). There were clearly COVID-19 induced impacts on access to international markets (see Table 1) but also an abalone factory was lost during the January bushfires, and there was a late start to the Western Zone Fishery. In addition, COVID-19 public health restrictions implemented in Melbourne affected the ability of some divers to travel around the state (see Table 1).

The southern rock lobster catch was very low during February 2020 due to COVID-19 induced impacts on international markets (Fig. 7b), but there appeared to be some recovery in May and June as was the case in South Australia and Tasmania.

Although a small fishery, wrasse catches were lower during February to April (Fig. 7c). This was due to the negative impacts of physical distancing and movement restrictions on the target live fish market, which serviced dine-in seafood restaurants in Melbourne (see Table 1). A similar impact was reported in Tasmania. Monthly catches of all other species and fisheries combined were slightly lower than the range of the previous five years during February to April and June. This was most likely due to a combination of COVID-19 induced impacts and management changes in some fisheries.

#### Western Australia

**Fig. 8:**
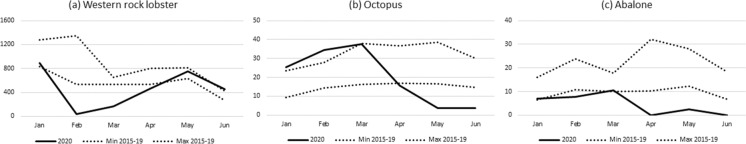
Monthly catch (tonnes) for (**a**) western rock lobster, (**b**) octopus, and (**c**) abalone in 2020, compared with the range of monthly catches for 2015–2019. *Source*: WA Department of Primary Industries and Regional Development

Catch records for the Western Rock Lobster Fishery, one of Australia’s most valuable fisheries, show a sharp reduction during February and March 2020, due to the loss of demand from and access to export markets in China caused by the COVID-19 outbreak and cancellation of key festivals (Fig. 8a) causing processors to stop taking product as no major markets were available. In response, the Western Australian Government later reduced the quota and extended the fishing season to ease pressure and give fishers longer to land quota. Monthly catches in the octopus fishery were lower from April to June (Fig. 8b). Fishing stopped due to a drop in domestic markets (e.g. restaurants closed due to physical distancing measures, see Table 1). Abalone catches were lower in February and from April to June (Fig. 8c). Similar to other jurisdictions this reflected COVID-19 impacts on international supply chains and distribution. However, catches were also reduced in some areas through management intervention due to sustainability concerns.

There was a combination of factors affecting production in other fisheries. In the Southern Coast Crustacean Fishery catches were low from January to May. Reduced catches resulted from reduced catch rates in parts of the fishery but also possible COVID-19 impacts due to supply chain disruption. Lower catches from February to June 2020 (excluding April) in the Joint Authority Demersal Gillnet and Demersal Line Fishery are attributed to lower effort due to a combination of less fishers fishing and COVID-19 induced impacts. For 2019–20, catches and effort were lower but catch rates remained stable, so changes in catch were not related to environmental factors or stock decline. In the Shark Bay Crab Fishery, the catch was low in February but overall the quota was met. However, there was a COVID-19 related staff shortage that affected factory operations early in the main fishing season but the company adapted.

For most other fisheries 2020 monthly catches sat in the range of the previous five years including those for prawns, scallops, squid and most finfish.

### Impacts on activities of fisheries research organisations

The impacts on fisheries research activities, field work and data collection varied between jurisdictions and organisations as well as over time from March onwards in response to government-led public health measures such as lockdowns, physical distancing, and travel restrictions. Differences in impacts reflected COVID-19 infection rates across Australian jurisdictions and the severity of public health measures implemented by each jurisdiction (see Table 1), as well as organisation-specific measures which reflected risk management systems and organisational structures. Organisations in jurisdictions with substantial Indigenous populations and with fisheries more severely impacted by trade shocks were also more impacted. Effects on activities are grouped into two broad areas: working arrangements, and field work, and assessment and management advice.

#### Working arrangements, travel and field work

During March and April most research staff (80–100%) in Australia’s fisheries research organisations were working from home with limited access to offices and laboratories. However, in the Northern Territory, all staff worked normally as researchers were identified as essential workers. Also, at the South Australian Research and Development Institute’s Aquatic and Livestock Sciences Division, while staff were initially encouraged to work from home, around 50% were working in the office during March and April. In New South Wales, Department of Primary Industry Fisheries Research staff were encouraged to work at home where possible, but research staff were considered essential workers and technical staff continued working at research sites and undertaking fieldwork while implementing COVID-safe work practises. An added complexity was agencies with multiple sites, regionally and across jurisdictions where different rules were in place. Online conference and meetings were widely adopted.

In May–June staff in about half of the organisations were transitioning back to the office as public health measures eased, although several still maintained 100% of staff working remotely. Strict COVID-19 safety protocols were adopted in workplaces, such as physical distancing, hand sanitisers, and additional cleaning services. Laboratory-based analyses and research activity still presented difficulties with the need for strict physical distancing. By September, staff at most agencies were working from the office or were transitioning back. Many retained flexible working conditions to meet physical distancing requirements through working part-time in the office, part-time at home.

Interstate and international travel ceased almost completely during this period and beyond accept in exceptional circumstances for essential travel. Initially, for most organisations, there was only limited travel within jurisdictions and usually limited to the local area and/or day trips only. Additional approvals and risk assessments were adopted to manage health risks arising through travel. In New South Wales, multi-day trips did occur subject to specific additional protocols. An issue raised during RPN discussions was the concern of regional communities was increased COVID-19 risks due to incoming intra-State travellers. Travel to Indigenous communities also ceased.

Travel in vehicles and vessels was also subject to physical distancing with limits on the number of travellers usually at one to two people for the former.

By June, restrictions on intra-state travel were easing although bans on travelling to Indigenous communities remained. By September intra-state travel was close to normal but additional travel approvals remained in place in some jurisdictions (e.g. Western Australia).

These travel restrictions, together with government-led physical distancing and movement restrictions, and organisational COVID-19 safety policies, had a significant impact on field work and data collection during March to June. This included fishery dependent and independent sampling. In addition, sampling was also impacted indirectly by effects on some fisheries and markets.

Impacts and operational responses varied across the period and between research organisations engaged in field work and are summarised in Table 4.

**Table 4:** Summary of the sequence of impacts to field work and operational responses reported by fisheries research organisations across the period January -June 2020

Research organisation	Sequence of impacts to field work and operational responses
Australian Fisheries Management Authority	From late March- to early June, no on-board observers were placed on domestic deployments, and onboard biomass surveys could not be undertaken. Limited Southern Ocean deployments and port sampling continued when possible By June, there was limited travel following approvals and some observers were being deployed
Commonwealth Scientific and Industrial Research Organisation (CSIRO)	Initially field work was reduced, and diving and small vessel activities suspended Research cruises on the Marine National Facility, *RV Investigator*, were also cancelled By June, field work within states was permitted along with diving and small vessel surveys
Institute for Marine and Antarctic Studies, University of Tasmania	Initially almost all field work was put on hold due to state government and university public health measures which limited the number of staff allowed on vessels and in vehicles. However, this was resolved after the first month including some commencement of field data collection such as processor sampling in modified form Diving surveys were rescheduled By late June, field-based activities were returning to normal although diving surveys were still paused Laboratory access remained a problem because while activities were occurring operational changes reduced the number of staff that could work in laboratories at any one time
New South Wales Department of Primary Industries	Within-state day trips continued, however there were restrictions on multi-day trips Multi-day trips that required accommodation other than camping were initially put on hold in response to concerns from regional communities about transmission risks Multi-day trips on large vessels were being undertaken where physical distancing was possible and hygiene standards increased Sampling at fishers’ cooperatives continued at some cooperatives as this could be undertaken by regional staff and contractors but was dependent on the manager of the cooperative Field and laboratory work, although reduced, was maintained throughout the period using Work Standards to accommodate COVID safety plan requirements for physical distancing for driving, diving, and boat-based activities
Northern Territory Department of Industry, Tourism and Trade	All onboard commercial vessel sampling trips were cancelled and observer coverage for harvest strategies was not undertaken Field trips were limited to daily trips and large field operations such as biomass surveys ceased Travel to Indigenous communities was not allowed Day trips on research vessels were allowed if physical distancing measures could be adhered to By June, full operations were resumed with travel to Indigenous communities and conducting onboard commercial monitoring permitted
Queensland Department of Agriculture and Fisheries	All non-essential travel stopped, and sample collection was only permitted less than 1.5 h from the office All onboard surveys stopped (including chartering vessels or going out on commercial vessels) and the annual fishery independent survey of Spanner Crabs was suspended Length and age data collection for 20 key species was suspended or reduced. These measures remained in place through to June State border closures also caused issues sampling some species, for example mullet in northern NSW In addition, fisheries monitoring had to adapt as new and changed markets affected sampling sites and methods
South Australian Research and Development Institute, Aquatic Sciences	On-board observers were not being deployed on surveys but data were obtained through commercial industry sources Intra-state vehicular trips (only day-trips initially until risk assessments were upgraded) were permitted and small vessel operations continued subject to appropriate risk mitigation The Research Vessel *MRV Ngerin* was berthed. In general, field work was assessed on a case-by-case basis; postponed, cancelled or progressed on the basis of a specific COVID-19 risk assessment that aligned with updated public health advice Overall, risk assessments were undertaken to determine field operations. In many cases field work to support fisheries assessment and management continued under COVID-19 risk mitigation strategies and were considered essential The cessation of fishing (e.g. Southern Rock Lobster Fishery) also affected data collection Laboratory analyses continued under strict physical-distancing arrangements
Victorian Fisheries Authority	Subject to approvals, most field work continued in Victoria. Length/age data were collected, and the Abalone Fishery independent survey was undertaken as scheduled. Fishery independent sampling for snapper, with small vessel-based teams, continued Southern Rock Lobster catch sampling was suspended
Western Australia Department of Primary Industries and Regional Development	Initially travel restrictions limited field work to local areas only with limited regional travel Research (monitoring and assessment, maintaining catch and effort data input etc.) in WA was deemed an essential activity where it was time-critical for ongoing resource management processes Field work was cancelled apart from priorities assessed on a case-by-case basis. For example, in-season prawn surveys were undertaken By May/June, field work was permitted except for work in the Kimberley region where there were large or highly vulnerable Indigenous communities Vessel-based activities were undertaken subject to risk assessments and subsequent approvals

#### Research organisations adaptive responses to mitigate COVID-19-induced disruptions

Clearly there was the potential for major impacts on fisheries monitoring, assessment and subsequent management advice. Data gaps and/or lower sample sizes, missed surveys due to travel restrictions, project delays and changed fishing fleet dynamics all had the potential to impact fisheries assessment.

During March-June 2020, fisheries research organisations across Australia adopted additional adaptive responses to help mitigate and adapt to the impacts of COVID-19-induced disruptions to their programs and activities. Some of these were in response to government-led policies and laws but others were primarily organisational measures. They can be broadly grouped into six categories: (1) managing new COVID-19 triggered risks, (2) modified data collection approaches, (3) maintaining staff productivity, (4) delivery of services and products to clients, and (5) strategic planning (Table 5). While our focus is on commercial fisheries some adaptive responses were also relevant for recreational fisheries assessment programs.

**Table 5:** Summary of initial countermeasures, risk management and continuity strategies by Australian fisheries research organisations in response to COVID-19-induced impacts

Organisation aims	Initial countermeasures, risk management and continuity strategies
Manage new COVID-19 triggered risks	Undertake risk assessments and audits of data collection and assessment activities Prioritise data collection activities Implement new risk management strategies (e.g. hygiene standards, social distancing) to allow high-priority activities to continue Schedule lab-based activities to manage risk and address priority needs IT systems enabling staff to work from home and other remote operations
Continue research and assessment activities using available means	Shift to staff working from home Shift to online/offsite version control and syncing of stock assessment files Shift to online meeting platforms
Collect data using available means	Adjust field trip plans and sampling programs and scope to manage assessed CV19 risks Use seafood processors, commercial and recreational fishers to collect, store and provide additional data and samples (e.g. freezing fish frames) Take advantage of regionalised staff distribution to reduce need for multi-day field trips Increase use of e-monitoring and remote data collection methods for monitoring commercial and recreational fisheries Change to (or increase proportion of) fisher survey methods using phone/online based
Maintain workforce productivity	Bring forward desk-based/low risk activities that require less field work and/or significant workshopping Transfer staff between activities (e.g. changing observer staff over to analysing e-monitoring footage)
Deliver products and services to clients	Re-schedule research projects to reflect assessed levels of CV19 risk in delivery Undertake additional assessments to assess impacts of management strategies addressing under catch/changed production across years)
Strategic planning	Review strategic priorities to align with industry/government needs during and post COVID and prepare for potentially lower research investment Introduction or increased reliance on flexible stock assessment methods which account for missing data points Revisit engagement strategies with less travel and continue the use of online conferencing platforms as appropriate Consider the potential for shared service between institutions and jurisdictions Use the RPN to develop national projects that align with industry/government needs in response to COVID-19 and its short and medium-term impacts

Risk assessments, audits and the prioritising of monitoring and assessment activities were undertaken, and new risk management measures implemented to enable high priority work to continue. Project portfolios were reviewed and projects re-prioritised as appropriate. Milestone delivery was assessed and, in some cases, renegotiated. These actions determined that most assessments undertaken during 2020 used data up to 2019 and were not affected. Two major research reporting activities—FRDC’s *Status of Australian Fish Stocks Reports 2020*, and ABARES *Fishery Status Reports 2020*—were undertaken with minimal disruption. For missed surveys that fed into management arrangements during 2020, organisations were able to mitigate risks by adopting approaches designed to account for the missing data points. Advice was also provided by fisheries research organisations to management agencies on the risks to stocks associated with quota roll-overs and other management measures.

Risks to future assessments conducted in 2021 and beyond were identified. Issues included the need to adopt agreed approaches for dealing with increased uncertainty in future stock assessment. Further issues included implications for Wildlife Trade Operation (WTO) accreditations for export approval required under the *Environment Protection and Biodiversity Conservation Act 1999* (EPBC Act), and the extent to which requirement for reporting and confidence in the quality of data concerning fishery interactions with Threatened, Endangered and Protected Species. Organisations also assessed and began to introduce measures to manage the extent to which very significant impacts on some fisheries would cause difficulties interpreting fishery dependent data, such as 2020 catch and effort statistics.

Risks to data sharing and project team connectivity were addressed by investment of considerable resources in upgrading Information Technology systems to allow the efficient working from home including onsite/offsite version control that enabled synchronisation of data files and documents. The rapid rate of adoption of these systems was mirrored in the agility observed by other fisheries research organisations globally (see Link et al. 2021). As was the case globally and in other sectors, there was rapid adoption of online platforms for meetings and workshops (Hacker et al. 2020). This meeting style was particularly effective for well-planned meetings and structured agendas. Negative outcomes from adopting online platforms for international meetings were reported (see Haas et al. 2021), arising from issues with time zones and multiple languages, band-width challenges, as well as for broad-ranging exploratory-style research workshops where open interaction between contributors is paramount. Face-to-face meetings were observed to still have an important role in meeting and workshop aims.

When data collection by field staff was not possible, industry participants and recreational fishers were used to collect and store samples for later analysis. The presence of regionally-located staff supported these strategies. Greater use of electronic monitoring was advocated including e-monitoring on commercial vessels and remote data collection methods for recreational fisheries such as boat ramp surveillance using CCTV and online survey methods. The strategy reported by many organisations of seeking alternate fishery-dependent data opportunistically was similar to those reported by other fisheries research organisations globally (see Huveneers et al. 2021; Santora et al. 2021).

Workforce productivity was maintained by bringing forward lower-risk desk activities such as data analysis and report writing. It was also possible to transfer staff between activities, for example field staff and observers were engaged in data entry and reviewing e-monitoring footage. Delivery to clients and funding agencies was maintained, as far as possible, through prioritising and rescheduling projects as required. New desk-based activities included research to assess risks arising from the implementation by governments of countermeasures to support fishing industries impacted by COVID-19 containment measures.

Alignment of organisational strategic priorities with changing fishing industry and government needs were reviewed by leaders of fisheries research organisations. Support for maintaining and using national coordination mechanisms, such as the RPN, for development of national projects addressing needs triggered by COVID-19 disruptions was reinforced. The need to develop strategies to respond to potentially lower levels of research investment by industry and governments in the short to medium-term was identified. The introduction or increased use of more flexible stock assessment methods which can account for short-term disruptions in data stream was also identified, as Link et al. (2021) similarly report for US fisheries.

In Australia it is not uncommon for research agencies to work across jurisdictional boundaries. Because of jurisdictional border closures (which varied over time) and differential approaches to fishery monitoring, the potential for shared services was discussed. Major constraints to these included restricted visitor access to laboratories and offices, different work health and safety policies and procedures and COVID-19 safety measures, and specific resourcing arrangements. However, the potential for shared services between organisations across jurisdictions was recognised and could perhaps be established under less disrupted conditions.

Many of the activities and responses described continued throughout 2020 and beyond following successive waves of COVID-19 variants.

## Conclusion

During the initial period of the COVID-19 pandemic from January 2020-June 2020 disruption to commercial fisheries production and fisheries monitoring and assessment activity in Australia was primarily due to pandemic-containment measures introduced by the Australian jurisdictions, as well as by governments in export-receiving countries. The containment measures were introduced to reduce the severity of the outbreak of the COVID-19 virus on public health. Consistent with global observations (FAO 2020), in Australia these measures directly affected fisheries harvesting, supply chain and research operations and activities. Less directly but more significantly, these measures led to changes in consumer behaviour and demand in seafood markets both domestically and internationally. While all Australian commercial fisheries production sectors and fisheries research organisations experienced disruptions, impacts observed on production levels and on monitoring and assessment activities are not uniform nor homogenous and reflect the extent of exposure to specific shocks and the extent of adaptive capacity.

Demand-side shocks to seafood markets were the more significant mechanism of disruption and impact to Australian fisheries production. The most evidently impacted fisheries are those for species sold live into export markets (for example, rock lobster and abalone). The responsiveness of production levels in these fisheries to market conditions is highlighted by the increase in production in May and June in once export markets started to re-open. Other impacted fisheries were those for species sold live or fresh into domestic dine-in food service markets (for example, coral trout, wrasse, crabs), where there was also a knock-on effect from competition with previously exported product being sold domestically. The shift in domestic consumer demand towards less perishable seafood purchased through retail channels introduced a positive disruption for some Australian fisheries sectors supplying these markets, although the impacts were observed on price rather than production levels directly. The disruptions to major export markets (i.e. China) and domestic markets (i.e. Melbourne and Sydney) appears to have uniformly impacted exposed Australian commercial fisheries sectors regardless of jurisdictional management arrangements.

Fisheries production in Australia did experience direct disruptions to harvesting and supply chain activity (for example, short-term labour shortages, restrictions on seafood processing facility operations, limited supply of long-haul cold chain freight services) due to domestic pandemic containment measures. However, these disruptions were less observable and more short-run. They were at least partly mitigated by national government countermeasures to protect food production activities and supporting services in these sectors from many restrictions through ‘essential worker’ policies. The range of countermeasures also included wage payment and business support, cost-relief measures, quota roll-overs and support for re-establishing freight links through the IFAM program, comparable with those implemented in other developed economies (OECD 2020).

In contrast to the commercial fisheries sector, Australia’s fisheries research organisations generally experienced disruptions triggered directly by government-led pandemic containment measures, combined with organisational COVID-19 risk management measures, and knock-on effects arising from impacts to commercial fishing levels. The degree of disruption and impact varied across jurisdictions and organisations, as in some jurisdictions research staff were deemed ‘essential workers’, although the types of disruption were broadly consistent with those reported by other fisheries research organisations globally (see Haas et al. 2021, Link et al. 2021; Santora et al. 2021).

Australia’s fisheries research organisations and their monitoring and assessment programs appear to have shown resilience and agility in the short-term under these conditions. The anticipated negative impacts on capacity and quality of assessment and management advice, at least in the short term, were less severe than originally anticipated. Adaptive strategies were broadly consistent with those reported by other fisheries research organisations globally. These included hastening the adoption of digital platforms enabling activities to continue online and remotely, and of integrating alternative forms of data and data collection.

In the case of fisheries production sectors, our analysis of this initial period highlights the vulnerability of those sectors with single markets, live product markets and/or long supply chains to these types of global systemic shocks (see Productivity Commission 2021). Further analysis over a longer time-period is warranted. Such analysis is required to take more full account of subsequent outbreaks of variants, the effects of both government countermeasures and industry adaptive responses (for example, re-directing rock lobster to the domestic markets or frozen product export markets), and confounding factors such as trade tensions, in order to assess the extent of recovery or residual impact on production under emerging production and market conditions. For fisheries research organisations, we echo Link et al. (2021) in concluding that examination is warranted of the extent to which these rapidly-implemented adaptive strategies implemented by fisheries research organisations will endure, and whether more structural innovations are required (such as further development of arrangements supporting cross-jurisdictional shared monitoring and assessment services). Nonetheless, what is reported in this paper provides important insights into the initial impacts and a baseline from which subsequent disruptions can be assessed.

## Data Availability

The data that support the findings of this study are available from the fisheries management and research organisations to which the authors are affiliated. But restrictions apply to the availability of these data, which were used under licence for the current study, and so are not publicly available. Data are however available from the authors upon reasonable request and with permission of fisheries management and research organisations listed, noting that requests will only be considered where they are consistent with commercial-in-confidence provisions within data policies of each data custodian.
